# Linking Helicobacter pylori with gallbladder and biliary tract cancer in Moroccan population using clinical and pathological profiles

**DOI:** 10.6026/97320630015735

**Published:** 2019-10-31

**Authors:** Soumia Cherif, Hanane Rais, Abdelmalk Hakmaoui, Souad Sellami, Said Elantri, Abdessamad Amine

**Affiliations:** 1 Department of Pathology, ARRAZI Hospital, Biopathology Laboratory-Clinical Research Center, Mohammed VI University Hospital, Marrakech, Morocco; 2 Laboratory of Biochemistry, Environment, and Agrifood, Faculty of Sciences and Techniques-Mohammedia, Hassan II University, Morocco/URAC-36; 3 Department of Gynecology, Charite University Medicine, Campus Virchow, Augustenburger Platz 1, 13353 Berlin, Germany

**Keywords:** Biliary duct malignancies, gallbladder malignancies, Morocco, histo pathological profile

## Abstract

It is of interest to assess the clinical and pathological aspects of Gallbladder and biliary tract carcinomas confirmed by histological data. It is also of further interest to evaluate the link between Helicobacter pylori and biliary tract cancers. Eighty-nine (89) cases (mean age 60±12 years) of Gallbladder and biliary tract cancer confirmed by histological data were enrolled for the study at the Department of Pathology in Mohammed VI University Hospital, in Morocco. The data such as age, sex, clinical and histo pathological features were collected. Bile duct specimens were investigated for H. pylori using Giemsa and immuno histo chemistry staining. Results show that bile duct stones were found in 53.9% of cases. It is known using histological data that adeno carcinoma is common accounting for 70 % of all bile duct tumors. Moreover, Helicobacter pylori was detected in 54% of cases linking with the presence of bile duct stones characterized by the histological subtype, the macroscopic classification and lymph node's presence (p<0.001). Thus, data collected suggest the potential association of Helicobacter pylori with gallbladder cancer possibly through the formation of bile duct stones.

## Background

Biliary tract, as well as gallbladder cancer, is aggressive, relatively rare and intractable malignancies. They are a heterogeneous group of neoplasms that emerge at any portion of the biliary tree in spite of their common tissue origin.Based on anatomical location, they include intrahepatic (IHCC) and extrahepatic cholangiocarcinoma (EHCC), gallbladder (GBC), and Ampulla of Vater carcinomas (AVC) [1].In the epidemiological databases, the worldwide incidence and mortality rates of bile duct cancers have changed since 1980 [1].Gallbladder carcinoma, which is the most common biliary tract malignancy and the sixth most common gastrointestinal cancer, exhibits a worldwide geographic variability, which can be explained by differences in environmental exposure and genetic predisposition [2].The highest rates are seen in Chile, Thailand, Japan, central Europe, Latin America, Northeastern India, and Pakistan but are rare in most regions of Europe and North America [3].Cholangiocarcinoma (CCA) account for only 3% of gastrointestinal malignancies there is increased mortality from intrahepatic cholangiocarcinoma and a decreased mortality in extrahepatic cholangiocarcinoma over the past several decades [4]. Moreover, carcinomas of the Ampulla of Vater accounts for 0.2% of gastrointestinal cancers. Compared with other biliary tract neoplasms, these tumors have a relatively favourable prognosis after surgical resection [5].

The incidence and pathological characteristics of carcinomas of the gallbladder and biliary tract in Morocco are largely unknown, although several investigations were performed. According to the Epidemiology Service of the National Institute of Oncology (NIO), 598 cases of the biliary tract and gallbladder cancer have been recorded between 1985 and 2002 in Rabat [6]. In Casablanca, 186 cases have been diagnosed between 2008 and 2012, and at Hassan II University Hospital in Fez 58 cases have been reported between 2004 and 2010 [7,8]. The etiology of biliary tract cancer remains unclear and not well understood. However, both epidemiological and experimental evidence has implicated several risk factors in the development of biliary tract and gallbladder cancer. In fact, these malignancies had been linked with the following factors: a history of gallstones, diet, obesity, ethnicity, primary sclerosing cholangitis, biliary-duct cysts and infectious agents [1,5,6,9]. Moreover, it has been suggested that a cause-effect association between Helicobacter spp. and biliary tract cancer is plausible. Helicobacter pylori (H. pylori) are a well-recognized causative factor of gastrointestinal diseases [10]. Several studies have investigated the implication of H. pylori infection in the extra-gastrique pathogenesis including benign and malign biliary diseases such as cholangiocarcinomas, gallbladder cancer, cholecystitis, and cholelithiasis [10-12]. In addition, to several toxins and metabolites delivered by H. pylori that might play a role in the biliary carcinogenesis [10,11]. Boonyanugomol et al. [13] demonstrated that cytotoxin-associated gene A pathogenicity island (cag PAI) is essential for H. pylori internalization in cholangiocarcinoma cells (CCA) with significantly reduced levels of NF-κB activation and IL-8 production by the same cells, thus H. pylori might play a causal role in the biliary pathogenesis. Moreover, Xiao et al. [14] performed a meta-analysis showing an association between Helicobacter species and cholangiocarcinoma. However, the exact carcinogenesis mechanism is still under debate [15]. In Morocco, and to our knowledge, there are no published data regarding the occurrence of these malignancies and their correlation with the presence of H. pylori. Therefore, it is of interest to assess the clinical and pathological aspects Gallbladder and biliary tract carcinomas confirmed by histological data. It is also of further interest to evaluate the link between Helicobacter pylori and biliary tract cancers.

## Methodology

### Study setting and population:

This retrospective study was conducted at the Department of Pathology in the University Center Hospital Mohammad VI (CHU) in Marrakech, Morocco. The study included 89 cases of bile duct cancer diagnosed over 14 years (January 2001 January 2015). Medical and pathology records of the bile duct and gallbladder cancer patients were retrieved and abstracted. Cases with insufficient clinical data were excluded. The histopathological examination was performed by 3 different pathologists and classified according to World Health Organisation (WHO) pathology and genetics of the digestive system (2010) [16]. After the macroscopic examination, the samples were formalin fixed and embedded in paraffin, sectioned, stained with HandE and then microscopically examined under an optical microscope. Only the patients for whom the diagnosis was confirmed by histology were included in the study. Since it's a retrospective study, no ethics approval is required. This study was performed in accordance with the principles of the Declaration of Helsinki.

### Data collection

Data collection was done retrospectively from patient records, using a standardized form. The collected variables included age, sex, clinical and histopathological data such as the presence of gallstones, preoperative diagnosis, macroscopic aspect, and anatomical location, the degree of invasion, histological type, and grade of differentiation. The collected data used to support the findings of this study are available from the corresponding author upon request.

### Detection of Helicobacter pylori

Detection of Helicobacter pylori was done by modified Giemsa staining. The confirmation was done subsequently by immunohistochemistry (IHC). A 4 µm section was cut from formalin fixed and paraffin embedded block, two different slides were prepared, which were stained by Modified Giemsa and anti-H.pylori antibody immuno stain.

### Modified Giemsa:

Paraffin-embedded sections were dewaxed and rehydrated. Then it is incubated for 20 min in 1:10 Giemsa's solution in distilled water. After rinsing in distilled water, the section was quickly dehydrated through alcohol solutions before being cleared with toluene and mounted.

### Immuno histo chemistry staining for H. pylori

For H. pylori detection, immunohistochemistry was performed on 4 µm paraffin sections and mounted on FLEX IHC Microscope Slides (Dako, United States). Briefly, sections were deparaffinized and re hydrated followed by heat-induced epitope retrieval using EnVision FLEX Target Retrieval Solution (Dako, United States). After blocking endogenous peroxidase with a 2% H_2_O_2_ solution, sections were incubated with the primary polyclonal rabbit anti- H. pylori antibody (Dako, United States) with a dilution of 1:10 at room temperature for 30 minutes. After sections were washed, a labelled-polymer- HRP (Dako, United States) was applied for 20 minutes. Finally, sections were incubated with 3, 3'-diaminobenzidine (DAB)+ substrate-chromogen (Dako, United States) for 10 min, and washed in tap water. Then, slides were immersed with hemato xylin and mounted. As a positive control, a gastric mucosal tissue infected with H. pylori was taken. 

### Statistical analysis

The ҳ^2^ or Fisher's exact test was used for statistical analysis to determine the relationships between the presence of H. pylori and categorical data, p < 0.05 was considered as statistically significant. The data was analysed by the SPSS V23 statistical program.

## Results

**Figure 1:**
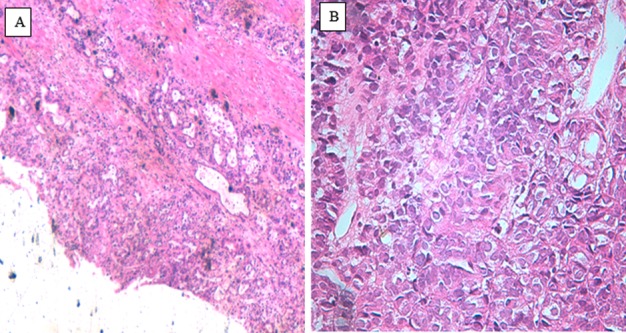
Histological types: (A) Adeno carcinoma in GBC (x40),(B) Adeno carcinoma in GBC

A total of 89 patients were identified with the diagnosis of gallbladder cancer (75 cases), intrahepatic (2 cases) or extrahepatic cholangiocarcinoma (8 cases) and Ampulla of Vater carcinoma (4 cases). The patient's characteristics are presented in Table 1. There were 67 females (76%) and 22 males (24%). The male to female ratios of different categories (GBC, IHCC, EHCC, and AVC) were 1:2.9, 1:1, 1:1 and 1.6:1, respectively. The mean age of all patients was 60±12 years old (range 21-90). Based on medical records, forty-eight patients presented biliary tract calculis consisting of mixed stones in twenty-four cases (50%), cholesterol stones in sixteen cases (33.3%) and pigments stones eight cases (16.7%). Macroscopically, the infiltrating type was the most common in different categories (83/89, 93.2%), followed by Ulcero-burgeoning (3/89, 3.4%), bourgeoning (2/89, 2.2%) and ulcero-infiltrating (1/89, 1.2%) type. Histological examination of tissue specimens (Figure 1) revealed that adenocarcinoma was the most common pathologic form, comprising 74.1% (66/89) of all cases. Other histologic types include papillary adenocarcinoma (9/89, 10.1%), Signet-ring cell carcinoma (4/89, 4.5%), adenosquamous carcinoma (3/89, 3.4%), mucinous carcinoma (3/89, 3.4%) and other histological types (4/89, 4.5%). The cell differentiation was divided according to predominant components: well differentiated (4/89, 4.5%), moderately differentiated (70/89, 78.6%), poorly differentiated (13/89, 14.5%) and undifferentiated (2/89, 2.2%). In term of loco regional extensions, lymph node metastasis was present in 14 cases of all categories.

**Figure 2:**
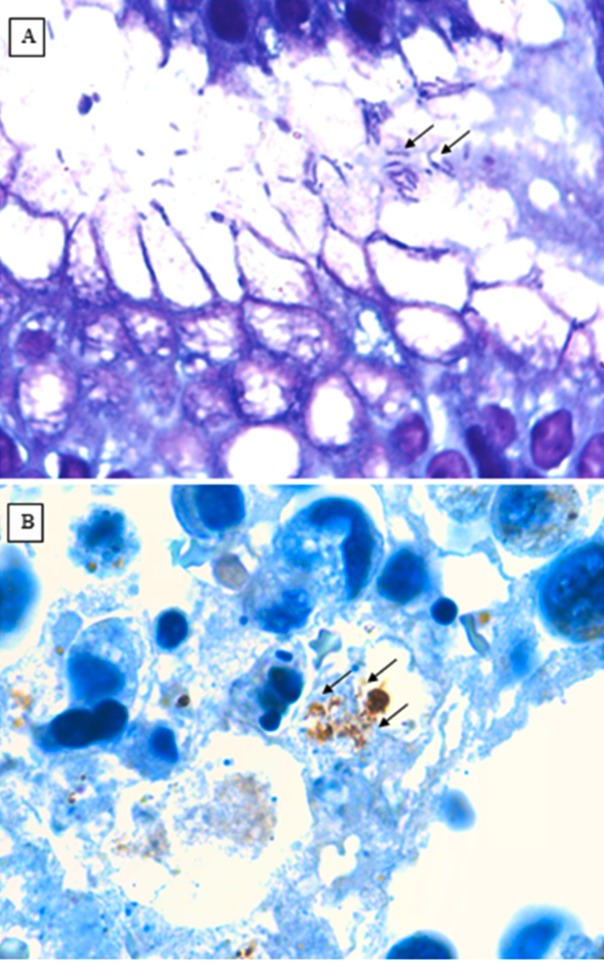
Histological staining techniques for Helicobacter pylori detection: (A) Giemsa stain showing H. pylori (x1000) and (B) Staining of H pylori polyclonal antibody by immuno histo chemistry (x1000).

After the examination by immunohistochemistry and Giemsa staining for H. pylori (Figure 2), 48 (54%) of the patients were positive and 41 (46%) of the patients were negative. Thirty-five were positive by H. pylori immunostain and Giemsa with the presence of calculis. There was a strong association between the presence of H. pylori and the presence of calculis (P<0.001). Moderately differentiated gallbladder and biliary tract cancer showing H. pylori positivity was present in forty-four. The p-value was <0.05 indicating a significant association between the presence of H. pylori and the differentiation of these malignancies, the histological subtype, the macroscopic variety and lymph node's presence. However, none of the demographic factors (gender and age), or the location of the tumor had a significant correlation with H. pylori (Table 2).

## Discussion

Gallbladder and biliary tract cancer is rare but carries a poor prognosis. Clinical and pathological evidence has suggested that inflammatory conditions involving the bile ducts predispose these ducts to carcinogenesis, however, the relationship between chronic inflammation and malignant transformation is still under debate [17,18]. These malignancies are difficult to diagnose in an early stage, the majority of patients develop symptoms only at an advanced stage of the disease. In term of tumor location, Gallbladder cancer is the most common malignancy in our investigation, representing 84.3% of all cases. Testerman et al. and Qu et al. reported similar findings [15,19]. Generally, biliary tract cancer has a peak incidence in the sixth and seventh decades of life and rarely occurs before the age of 40 [19-22]. The average age in our study was 60 years old. Comparable results were mentioned in other studies carried out in Casablanca and Fez [6,8].

GBC is seen commonly in women. In contrast, CCA is marked with a male predominance with a ratio of 1:1.2-1.5 [6,18,23].It has been suggested that bile duct stones and their characteristics such as the size and the type are considered as an important risk factor for these tumors [2,5]. In our study, Forty-eight patients presented biliary tract calculis consisting of mixed stones 24(50%), cholesterol stones16 (33.3%) and pigments stones 8 (16.7%). The formation of stones predisposes to biliary carcinogenesis by causing chronic mucosal damage due to mechanical forces, and induces in a local production of carcinogen such as secondary bile acids [2]. Histopathologically, adenocarcinoma was the most common histological type found in this study. The prognosis of adenocarcinoma of the gallbladder is strictly related to the grade of local diffusion and to the possibility of an R0 resection of the tumor [24].

Epidemiological and clinical evidence indicate that H. pylori might be an etiological factor for CCA and GBC especially in the regions with a higher prevalence of this infectious agent [10]. Chen DF and al., showed that H. pylori induces apoptosis in human gallbladder epithelial cells which can be explained by the implication of this pathogen in the activation of factors inhibiting cell proliferation, promoting the expression of regulatory genes like bcl-2, bax and finally inducing cell apoptosis [25]. In the biliary tract, H. pylori were detected in gallstone disease [26]. It was also associated with viral hepatitis, primary biliary cholangitis and primary sclerosing cholangitis [27]. However, studies conducted so far showed a high variability in methods and also in findings. In some studies a strong association between H. pylori and biliary tract diseases was reported, while others failed to find a statistically significant association [28-30]. Furthermore, some studies have examined the link between H. pylori infection and cholecystitis, gallbladder cancer, but results have been inconsistent [31-43].

In the present study, H pylori were detected in 54% of patients and this infectious agent was associated with calculis, the differentiation of the tumor, the histological subtype, the macroscopic variety and lymph node's presence. A positive association between H. pylori infection and gallstones in humans was also reported by analyzing a large cohort [44]. Moreover, Griniatsos J et al., suggest that H. pylori are related to gallstone formation [45]. Li et al. mentioned that H. pylori were 9.9 times more frequent in patients with biliary tract carcinoma compared with patients in the control group [46]. More importantly, a study conducted in Thailand demonstrated that in patients with CCA, a significantly high inflammatory grade and cell proliferative index in the H. pylori PCR-positive samples in comparison to the H. pylori negative ones [11]. These reports provide strong integrity to the hypothesis that Helicobacter is associated with biliary cell inflammation and proliferation.

Although, several pathways by which H. pylori might induce a cancerous change in bile duct has been proposed. One mechanism called "the peri genetic pathway" involves the enhanced production of free radicals close to H. pylori and an increased rate of host cell mutation. It has been proposed that Helicobacter induces inflammation and locally high levels of tumor necrosis factor-alpha (TNF-α) and interleukin-6. According to the anticipated peri genetic mechanism, signaling molecules associated with inflammation, such as TNF-α, alter epithelial cell adhesion and lead to the dispersion and movement of mutated epithelial cells without the need for additional mutation in tumor suppressor genes [47].

## Conclusion

It is of interest to confirm the presence of H. pylori in Gallbladder and biliary tract cancers using clinical and pathological data. The results link H. pylori with the development of CCA and GBC. Thus, Helicobacter infection in CCA and GBC patients confirmed with histopathological data implies its association with the bile duct stone formation. Moreover, the association between H. pylori infection and the degree of differentiation of Gallbladder and biliary tract cancer is also ascertained. However, additional data is needed to evaluate the link between the carcinogenesis of H. pylori in the biliary tract using in vitro and in vivo models. Data on the risk factors of these malignancies including Helicobacter infections, gallstones, environmental factors, genetic susceptibility and possible regional differences is also relevant.

## Figures and Tables

**Table 1:** Clinical and histo pathological characteristics of different categories of biliary tract and gallbladder cancers (N= 89)

Variable (n/N)	GBC (n=75)	AVC (n=4)	IHCC (n=2)	EHCC (n=8)
Sex
Female (67/89)	56 (74.6%)	2(50%)	1(50%)	3(37.5%)
Male (22/89)	19 (25.4%)	2(50%)	1(50%)	5(62.5%)
Age	60±12.04	58.7±11.1	74±11.2	63±10.1
Calculis				
Presence of calculis(48/89)	48 (64%)			
Absence (27/89)	27 (26%)			
Types of calculis				
Mixed stones (24/48)	24/48(50%)			
Cholesterol stones (16/48)	16/48 (33.3%)			
Bilirubin stones (8/48)	8/48 (16.7%)			
Histological types				
Adenocarcinoma(66/89)	58 (77.4%)	2 (50%)	2(100%)	4(50%)
Papillary adenocarcinoma (9/89)	6 (8%)	2 (50%)		1(12.5%)
Signet-ring cell carcinoma (4/89)	2 (2.7%)			2(50%)
Adenosquamous carcinoma (3/89)	3(4%)			
Mucinous carcinoma (3/89)	3(4%)			
Cystadenocarcinoma (2/89)	1(1.3%)			1(12.5%)
Squamous cell carcinoma (1/89)	1(1.3%)			
Carcinoid tumor (1/89)	1(1.3%)			
Differenciation				
Moderately (70/89)	64 (85.3%)	1(12.5%)		5(62.5%)
Poorly (13/89)	10 (13.3%)	2(50%)		1(12.5%)
Well (4/89)	1(1.3%)	1(12.5%)	1(50%)	1(12.5%)
Undifferentiated (2/89)	0(0%)		1(50%)	1(12.5%)
Lymph node				
Presence (14/89)	8(10.6%)	1(12.5%)	1(50%)	4(50%)
Absence (75/89)	67(89.4%)	3 ( 75%)	1(50%)	4(50%)
Macrosopic varieties				
Infiltrating type (83/89)	72 (96.1%)	2(50%)	1(50%)	8 (100%)
Ulcero-burgeoning type (3/89)	1(1.3%)	1(12.5%)	1(50%)	
Burgeoning type (2/89)	1(1.3%)	1(12.5%)		
Ulcero-infiltrating (1/89)	1(1.3%)			

**Table 2:** Characteristics of the patients linked with H. pylori infection (N=89) Chi square (χ^2^) test

Variable	HP+	HP-	P-value
Sex
Female	36	31	0.94
Male	12	10	
Age			
≥50	8	7	0.95
≤51	40	34	
Calculis			
Presence of calculis	35	13	<0.05*
Absence	13	28	
Histological types			
Adenocarcinoma	46	20	< 0.05*
Papillary adenocarcinoma	0	9	
Signet-ring cell carcinoma	1	3	
Adenosquamous carcinoma	0	3	
Mucinous carcinoma	0	3	
Cystadenocarcinoma	1	1	
Squamous cell carcinoma	0	1	
Carcinoid tumor	0	1	
Differenciation			
Moderately	44	26	< 0.05*
Poorly	1	12	
Well	3	1	
Undifferentiated	0	2	
Lymph node			
Presence	8	0	<0.05*
Absence	40	41	
Macrosopic varieties			
Infiltrating type	48	35	0.06
Ulcero-burgeoning type	0	3	
Burgeoning type	0	2	
Ulcero-infiltrating	0	1	
Location			
GBC	44	31	0.12
AVC	2	2	
IHCC	0	2	
EHCC	2	6
